# Encapsulation of Pomegranate Peel Extract (*Punica granatum* L.) by Double Emulsions: Effect of the Encapsulation Method and Oil Phase

**DOI:** 10.3390/foods11030310

**Published:** 2022-01-24

**Authors:** Leyla Sanhueza, Paula García, Begoña Giménez, José Manuel Benito, María Matos, Gemma Gutiérrez

**Affiliations:** 1 Departamento de Ciencia de los Alimentos y Tecnología Química, Facultad de Ciencias Químicas y Farmacéuticas, Universidad de Chile, Santiago 8380000, Chile; leyla.sanhueza@ug.uchile.cl; 2 Departamento de Nutrición, Facultad de Medicina, Universidad de Chile, Santiago 8380453, Chile; pgarcia@uchile.cl or; 3 Departamento de Ciencia y Tecnología de los Alimentos, Facultad Tecnológica, Universidad de Santiago de Chile, Santiago 9170124, Chile; bego.gimenez@usach.cl; 4 Department of Biotechnology and Food Science, University of Burgos, Plaza Misael Bañuelos s/n, 09001 Burgos, Spain; jmbmoreno@ubu.es; 5 Department of Chemical and Environmental Engineering, Institute of Biotechnology of Asturias, University of Oviedo, 33006 Oviedo, Spain; matosmaria@uniovi.es

**Keywords:** double emulsion formulation, punicalagin encapsulation, emulsion stability, oil viscosity, membrane emulsification

## Abstract

Pomegranate peel is an agro-industrial waste that can be used as source of punicalagin, a polyphenolic compound with several beneficial effects on health. Since, once extracted, punicalagin is prone to degradation, its encapsulation by double emulsions can be an alternative to protect the active compound and control its release. The aim of this investigation was to evaluate the feasibility of encapsulating pomegranate peel extract (PPE) in double emulsions using different types of oils (castor, soybean, sunflower, Miglyol and orange) in a ratio of 70:30 (oil:PPE) and emulsification methods (direct membrane emulsification and mechanical agitation), using polyglycerol polyricinoleate (PGPR) and Tween 80 as lipophilic and hydrophilic emulsifiers, respectively. Direct membrane emulsification (DME) led to more stable emulsions during storage. Droplet size, span values, morphology and encapsulation efficiency (EE) were better for double emulsions (DEs) prepared by DME than for mechanical agitation (MA). DEs formulated using Miglyol or sunflower oil as the oily phase could be considered as suitable food grade systems to encapsulate punicalagin with concentrations up to 11,000 mg/L of PPE.

## 1. Introduction

In the last decade, interest in pomegranate fruit (*Punica granatum*) has increased since its consumption is associated with preventive and attenuating activities against numerous chronic and health/life-threatening maladies such as cancer, type 2 diabetes, atherosclerosis and cardiovascular diseases [1]. Pomegranate is consumed as fresh arils or as processed products, e.g., fresh or concentrated juice, infusions or jam. Nevertheless, juice yield is only the 40% of the whole fruit, and the remaining corresponds to pomegranate by-products considered wastes such as pomegranate peel (PP), seeds and mesocarp [2].

Food loss and waste generates an environmental, social and economic impact [3]. For this reason, the 2030 agenda for the United Nations sustainable development goals (SDGs) set food waste reduction targets (SDG 12) [4]. PP is considered a by-product or waste without commercial value. However, several studies have shown it contains phenolic compounds such as phenolic acids, flavonoids and hydrolysable tannins [5]. These last are known as ellagitannins, with punicalagin being the most important compound [6]. Antimicrobial, antiviral, anticancer, antioxidant and antimutagenic properties have been described for punicalagin in vitro and animal models [7,8,9,10]. Therefore, PP or pomegranate peel extract (PPE) could be potential new food additives, reducing waste in the agri-food industry [11,12].

Once extracted from PP, phenolic compounds are prone to degradation due to adverse environmental conditions. Moreover, when they are incorporated into food, they could produce off flavors and aromas. Therefore, encapsulation is a suitable option to stabilize the phenolic compounds, wherein the active compounds are entrapped in a polymeric matrix to protect them and mask flavors and control their release [13].

PPE has been encapsulated by different methods, such as spray drying, ionic gelation and pickering emulsions by several authors [7,13,14,15,16,17,18,19]. However, the PPE encapsulation by double emulsions (DEs) is not frequently studied. Water-in-oil-in-water (W_1_/O/W_2_) DEs are systems in which droplets of a water-in-oil (W_1_/O) emulsion are dispersed within an external continuous aqueous phase (W_2_). One advantage of DEs as an encapsulation method is that their multicompartmentalized structure allows for a controlled release of the encapsulated bioactive compounds from the internal to the external aqueous phase. Furthermore, DEs may protect sensitive bioactive compounds from degradation and mask undesirable sensory properties that certain bioactive compound may have [20]. In spite of these advantages, the stability control of DEs could be a problem with regard to the shelf life of a product. This instability usually leads to the loss of a significant part of the internal phase as early as during the production of the DEs. In general, the formulation of stable DEs becomes a difficult issue due to their large interfacial area that requires the use of at least two types of stabilizers. Therefore, it is necessary to optimize the production process [21,22].

DEs can be formed by one- or two-step emulsification processes, the latter being more used. In the first stage, a W/O emulsion is prepared with a hydrophobic emulsifier by strong homogenization to form tiny droplets, typically less than 5 microns. In the second stage, a W/O/W double emulsion is further formed by gentle addition of the W/O (termed inner emulsion) to a second water phase in which hydrophilic emulsifier has been dissolved. Various emulsification methods such as mechanical agitation (MA), high pressure homogenizers, membrane emulsification (ME) and other membrane-based methods have been reported [22,23], and the membrane-based methods have been reported to give narrower droplet size distributions. ME could be divided into two different types: direct membrane emulsification (DME) and premix membrane emulsification. DME is based on the injection of the dispersed phase through the membrane pores, and the growing droplets are dragged by the continuous phase, while premix ME is based on a two-step process in which firstly a coarse DE is prepared by mechanical agitation, and then it is passed through a membrane in order to reduce and homogenize the droplet size [22,24,25]. In this study, DME was used because this technique is highly attractive given its simplicity, potentially lower energy demands, low stabilizer concentration required and the resulting narrow droplet size distributions on the emulsions formed [25].

The aim of this work was to evaluate the feasibility of encapsulating PPE with high punicalagin content by the DE method. The second step for DE production will be MA and DME, using several oils as the oily phase. DEs prepared were characterized in terms of droplet size, droplet size distribution, morphology, stability and encapsulation efficiency (EE).

## 2. Materials and Methods

### 2.1. Materials

Pomegranate fruits (cv. Wonderful) were collected at the ripening stage (April 2017) from a commercial farm located in Vallenar (28°SL), in the Atacama Region of Chile. Fruit samples were stored at 4 °C and processed within 24 h of collection. Orange oil (density 843 kg/m^3^ at 25 °C), castor oil (density 836 kg/m^3^ at 25 °C), Miglyol^®^ 812 (density 945 kg/m^3^ at 20 °C), sunflower oil (density 919.3 kg/m^3^ at 25 °C) and soybean oil (density 850 kg/m^3^ at 25 °C) were supplied by Sigma–Aldrich Co. (Darmstadt, Germany). Polyglycerol polyricinoleate (PGPR, C_21_H_42_O_6_) was purchased from Brenntag AG (Essen, Germany) and used as a lipophilic emulsifier. Tween 80 (polyoxyethylene sorbitan monooleate), supplied by Sigma–Aldrich Co. (Germany), was used as hydrophilic emulsifier. HPLC-grade acetonitrile, methanol and chloroform were obtained from Sigma–Aldrich (St. Louis, MO, USA).

### 2.2. Sample Preparation

PP from fresh fruits was manually separated and dried by convection in an air-drying tunnel (no brand, built with a Tetlak motor) with a horizontal air flow rate of 2 m/s and 50% recirculation at 60 °C for 16 h. The dried product was ground in a knife mill (Wiley Mill, Model−2, A.H. Thomas Co., Philadelphia, PA, USA) to obtain a particle size of 20 mesh. The resulting pomegranate peel powder was stored in darkness and kept at room temperature until extraction.

### 2.3. Pomegranate Peel Extract Preparation

PPE was obtained using a conventional solid–liquid extraction in a solvent: peel ratio of 10:1. Pomegranate peel powder sample was treated with ethanol:water (40:60 *v/v*) with stirring at 159 rpm for 3 h. Then, the extract was centrifuged at 4000× *g* for 15 min, and the solid material was filtered out through a filter paper under vacuum. Ethanol was removed from the extract with a rotary evaporator (R-100 Büchi, Flawil, Switzerland) at 40 °C. Optimal conditions of pomegranate peel extraction were previously established by experimental design (data not shown).

### 2.4. Water-in-Oil (W_1_/O) Emulsions Preparation

First, W_1_/O single emulsions were prepared using 30% (*w/w*) of the inner aqueous phase with PPE (W_1_) and 70% (*w/w*) of the continuous oily phase (O). The internal aqueous phase (W_1_) was an ethanolic solution containing the PPE and NaCl 0.01 M. Oil containing 6% (*w/w*) of hydrophobic emulsifier (PGPR) previously dissolved by stirring at 50 °C for 30 min was used as the continuous phase, since it was found to be an appropriate emulsifier to stabilize W_1_/O emulsions [26,27]. Both continuous and dispersed phases were emulsified in glass vessels by high shear mixing (Silentcrusher M Homogenizer, Heidolph, Schwabach, Germany) using a 6 mm dispersing tool at 20,000 rpm for 5 min [28,29]. Five different types of W_1_/O emulsions were prepared using different oils, such as castor oil, soybean oil, sunflower oil, Miglyol and orange oil, keeping constant the concentration of PGPR and the W_1_.

### 2.5. Water-in-Oil-in-Water (W_1_/O/W_2_) DE Preparation

#### 2.5.1. Preparation of DEs by Mechanical Agitation (MA)

The W_1_/O/W_2_ DEs were prepared by MA using a rotor–stator homogenizer (Silentcrusher M Homogenizer, Heidolph, Schwabach, Germany) provided by a 6 mm dispersing tool at 10,000 rpm for 3 min. A total of 20 mL of W_1_/O were prepared with a ratio of 20:80 of W_1_/O into the W_2_ phase [29]. The external aqueous phase contained 2% (*w/w*) of hydrophilic emulsifier (Tween 80), previously dissolved by stirring for 30 min, as was established by Matos and coworkers in previous studies [29].

NaCl 0.01 M was also added to the external phase to balance the osmotic pressure between the two aqueous phases [29].

#### 2.5.2. Preparation of DEs by Direct Membrane Emulsification (DME)

W_1_/O/W_2_ DEs were produced by DME, composed of a two-step emulsification process. First, a W_1_/O emulsion was prepared by MA following the procedure described in Section 2.5.1. Then, the W_1_/O emulsion was injected through the bottom into a 200 mL stirred batch ultrafiltration cell Amicon model 8200 (Millipore, Billerica, MA, USA) which was used for DME experiments. This cell was equipped with a 5 µm pore size nickel membrane, supplied by Micropore Ltd. (Derbyshire, UK), with a distance between pores of 200 µm and a thickness of 200 µm. The membrane was previously soaked in the continuous phase [30,31].

The primary emulsion (W_1_/O) was forced into the cell by a syringe pump KDS-100-CE (KD Scientific, Holliston, USA) at a rate of 20 mL/h. The continuous phase (W_2_) in the upper part of the cell where the primary emulsion was injected through the membrane was continuously agitated at 400 rpm [30,32]. Membranes were cleaned by the use of an ultrasonic bath (Selecta, Barcelona, Spain, 110 W of power): first with a dishwashing detergent and deionized water for 10 min, followed by acetone for 15 min. Finally, the membrane was dried using compressed air and soaked in the continuous phase, as was established by Matos and coworkers in previous studies [27,31].

### 2.6. Viscosity Measurement of Oils

The viscosity of the vegetable oils was determined using a MARS II rotational rheometer (Haake, Karlsrughe, Germany). All the analyses were carried out at 25 °C using a plate/plate system (PP35) with a gap of 1 mm. Samples rested for at least 5 min previous to any measurement, allowing the stresses induced during sample load to relax. All measurements were performed in triplicate, and the data were processed by the Haake Rheowin 4.0 Software [28].

### 2.7. Characterization of DEs

#### 2.7.1. Droplet Size Distribution (DSD)

Droplet size distributions (DSD) of the W_1_/O/W_2_ DEs were measured by laser diffraction using Malvern Mastersizer S long bench equipment (Malvern Instruments Ltd., Malvern, UK) in fresh DEs and after 20 days of storage at 25 °C applying the methodology used in previous works by Gutiérrez and coworkers [33]. The polydispersity of the droplet size of the DE was expressed in terms of span, Equation (1):

(1) $$\mathrm{Span}=\frac{D\left(v,0.9\right)\;-\;D(v,0.1)}{D(v,0.5)}$$

where D(v,0.9), D(v,0.5) and D(v,0.1) are standard percentile readings from the analysis. They correspond to diameters at which 90%, 50% and 10% of droplets volume are of smaller size, respectively. Lower span values are associated with narrower size distributions [28].

#### 2.7.2. Microscopic Studies

Micrographs of the DEs were obtained with a light microscope Olympus BX50 (Olympus, Tokyo, Japan) with 40× magnification, in fresh DEs and after 20 days of storage.

#### 2.7.3. Stability

The stability was determined by measuring backscattering (BS) and transmission (TS) profiles in a Turbiscan apparatus (Formulaction, L’Union, France). Emulsions were placed without dilution in the test cells. BS and TS light were taken from 20 mL of sample every 24 h for 20 days [29,30].

#### 2.7.4. Encapsulation Efficiency (EE)

The EE of DEs was estimated by measuring the concentration of punicalagin in the W_2_ phase, as was stablished in several works by different authors [9,34,35]. For this purpose, DEs were centrifuged (2500× *g*, 30 min) to separate oil globules from the W_2_, filtered (0.22 µm, Millipore filter), and then W_2_ injected were analyzed into the RP-HPLC (P_W2_), according to previous studies [36,37].

**Figure 1:**
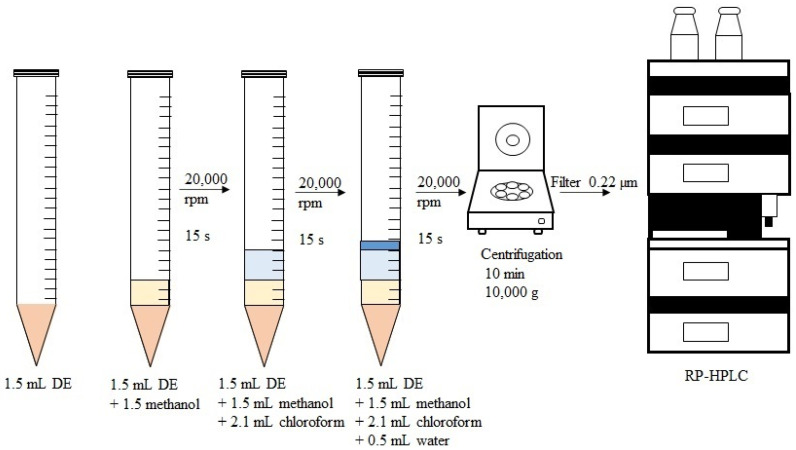
Schematic representation for determination of total punicalagin content in the DE.

Additionally, to measure the total amount of punicalagin in the prepared DEs, encapsulated and nonencapsulated, 1.5 mL of DEs were homogenized sequentially with methanol (1.5 mL), chloroform (2.1 mL) and distilled water (0.5 mL) by using a rotor–stator (Silentcrusher M Homogenizer, Heidolph, Schwabach, Germany) at 20,000 rpm for 15 s (each solvent) to ensure DE rupture and, hence, liberation of the encapsulated punicalagin. Then, samples were centrifuged at 10,000× *g* for 10 min, filtered (0.22 µm, Millipore filter) and injected into the RP-HPLC (Pt), (HP series 1100 chromatograph, Agilent Technologies, Inc., Santa Clara, CA, USA). The process is schematically described in Figure 1. The system was equipped with a UV-vis absorbance detector HP G1315A or a fluorescence detector 1260 Infinity A (Agilent Technologies, Inc., Santa Clara, CA, USA), Zorbax Eclipse Plus C18 column of 5 µm particle size and 4.6 mm × 150 mm (Agilent Technologies, Inc., Santa Clara, CA, USA).

The mobile phase consisted of a mixture of (A) 0.4% acetic acid in Milli-Q water and (B) 100% acetonitrile with gradient elution at a flow rate of 1 mL/min. The step gradient started with 95% mobile phase (A) running 95% of mobile phase (B) in minute 37.

EE was calculated by the following equation:

(2) $$\mathrm{EE}=100-\frac{P_{w2\;}\times 100}{P_{t}}\cdot {\;F}_{p}\;$$

where F_p_ (Equation (3)) corresponds to a correction factor that considered the punicalagin content which could get lost during agitation or that could interact with solvents during the process used to determine total punicalagin content. This factor prevents an overestimation of EE due to the nonencapsulated punicalagin lost during the process.

For this purpose, a standard emulsion, where 100% of W_1_ is present in W_2_, was formulated. Therefore, an oil-in-water (O/W_2_) emulsion was prepared using the same formulation as in the other experiments. Then, this O/W_2_ emulsion was diluted at the same ratio with W_1_, which contained the appropriate amount of punicalagin initially added (P_t_). Punicalagin content in the external aqueous phases (P_wt_) was determined experimentally by RP-HPLC after centrifugation and filtration, as it was aforementioned. Additionally, P_t_ was calculated for each sample. A value for F_p_ of 1 will indicate that the determination and preparation processes do not imply any punicalagin lost. The punicalagin content was measured in fresh DEs and after 20 days of storage.

(3) $$F_{p}=\frac{P_{\mathrm{wt}}}{P_{t}}$$

### 2.8. Statistical Analysis

The differences in the analyses were realized using a one-way and multifactor ANOVA test for means comparison, depending on the case. When significant differences were found, the Tukey HSD (honest significant differences) multiple-comparison test (*p* ≤ 0.05) was applied. Analyses were performed with Statgraphics Centurion XV, Version 15.1.02 (StatPoint, Inc., Warrenton, VA, USA).

## 3. Results

### 3.1. Viscosity Measurements of the Oils

The effect of the viscosity of the oil phase on the stability of DEs was studied. Viscosity is an important parameter influencing preparation, stability of multiple emulsions, encapsulation capacity and biocompound release, because the oil phase could control the rate of solute release through the oil membrane [38,39]. Table 1 shows the viscosities of the five oils used for DE formulation. Castor oil showed the highest viscosity among the oils tested, while Miglyol and orange oil showed the lowest values, which were similar.

### 3.2. Droplet Size Distribution (DSD)

The droplet size has been considered as control and comparison criterion for DEs prepared by several formulations using different preparation methods. It is commonly assumed that the smaller the droplet size, the better the stability of the emulsion, particularly versus creaming and clarification phenomena [31]. Moreover, the effect of time on droplet size has been studied by measuring DSD for fresh samples and after 20 days of storage at 25 °C.

**Figure 2:**
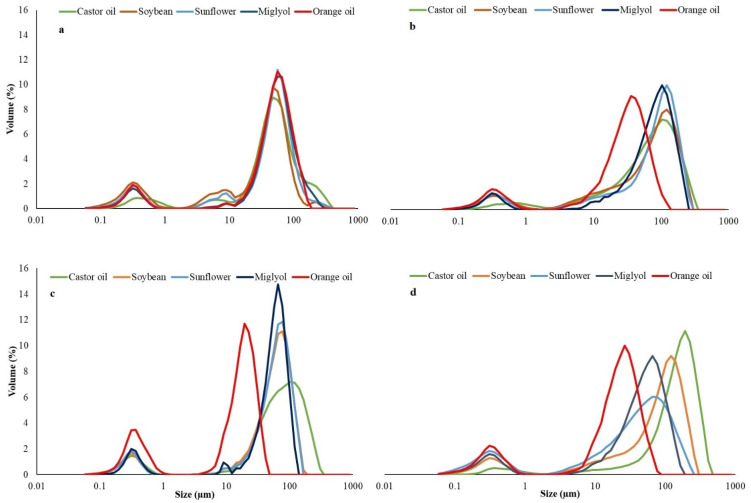
Droplet size distribution in the DE. (**a**) Fresh DEs prepared by DME. (**b**) DEs prepared by DME after 20 days of storage. (**c**) Fresh DEs prepared by MA. (**d**) DEs prepared by MA after 20 days of storage.

DSD for fresh and 20-day-old samples is presented in Figure 2. All DEs formulated with different oils showed a bimodal size distribution. For the first population, the droplet size ranged between 0.1 and 1 µm, whereas sizes in the range 8–800 µm were observed for the second peak in both fresh and 20-day-old samples.

Previous studies by Matos et al. [27] reported bimodal droplet size distributions with similar droplet sizes in DEs prepared by DME using Miglyol as the oil phase, 5% PGPR and 2% Tween 20. DEs prepared by MA with 20/80 ratio of W_1_/O in W_2_ also showed a bimodal droplet size distribution similar to the one obtained in the present study, indicating that the small peak could correspond to light scattered by the inner droplets [29]. However, the same behavior has been observed by Smulek et al. [40] when nanoemulsions stabilized by proteins were prepared. This behavior was observed when higher proteins concentrations were used, indicating that the presence of this small peak could be due to the presence of some agglomerates of proteins or lipophilic surfactants in the present study.

Droplet size and DSD in DEs is largely influenced by the processing conditions, the type of oil phase, the viscosity of the phases, presence of bioactive compounds, composition, the type of emulsifier used and the concentration of dispersed phase used. Usually, low oil concentration leads to smaller droplet size, as observed in Jarzebski et al. [41]. Therefore, it is difficult to compare the results among studies when considering the variations of these parameters [21,42].

Most of the samples prepared by MA showed larger droplet sizes than those obtained by DME (Figure S1, supplementary material), since, in conventional emulsification processes, high-shear stresses are needed to decrease the droplet size and must be considered according to the viscosity of the oil phase [22]. It is important to note that DME normally led to droplet sizes of around 2–10 times the membrane pore diameter used [25,32]. However, in this study, larger ratios were found, as it has also been similarly reported by Yuan et al. in previous studies using metallic membranes [43].

Table 2 shows mean droplet sizes, referred to as the mean value of the main peak, and span of all DEs prepared. The DEs produced by DME using orange oil as the oily phase showed a reduction in size over time. However, the DEs prepared with the other oils showed either constant or larger size with time, the latter attributed to coalescence of the oil drops. The reduction in size found when orange oil was used is a less common behavior in emulsions with storage time. However, in the case of DEs, it is observed when W_1_ water drops migrate from the oil phase to the external W_2_ [44,45].

As a general trend, the DEs prepared by DME showed lower size variations, resulting, therefore, in more stable and more controlled droplet size, in agreement with previous studies [22,27].

The highest variation in the polydispersity (span) during storage was found in the samples prepared by MA, which is consistent with results in Figure 2, where an increase in polydispersity can be observed in all cases, except for DEs formulated with castor oil. This effect is produced because of droplet coalescence and Ostwald ripening phenomena, in which small droplets disappeared to produce larger droplets, increasing the droplet size and the polydispersity at same time (Table 2).

Table 2 shows that there were significant differences (*p* < 0.05) in the polydispersity of fresh DEs between methods (MA vs. DME), but those differences were not appreciable after 20 days of storage, since polydispersity was statistically the same between both methods used after 20 days. On the other hand, in fresh DEs, there were significant differences among oils when they were prepared by MA, and even larger differences were observed when they were prepared by DME. Moreover, the polydispersity of the emulsions prepared with castor oil was significantly higher compared to those produced with orange oil, indicating that low viscosity oils could produce narrower size distributions. Previous studies by Yuan et al. show that oil viscosity is one of the key parameters on oil drop rupture during the MA process or on the detachment of oil drops from a membrane surface in the DME process [43].

It should be noted that the simple ANOVA between the different days (for each method) showed that there were no significant differences in polydispersity over time in the case of the emulsions prepared by DME; this is in contrast with emulsions prepared by MA, where significant differences were found in polydispersity with storage time, confirming that emulsions prepared by DME were less affected by destabilizing phenomena than those prepared by MA.

### 3.3. Morphology

**Figure 3:**
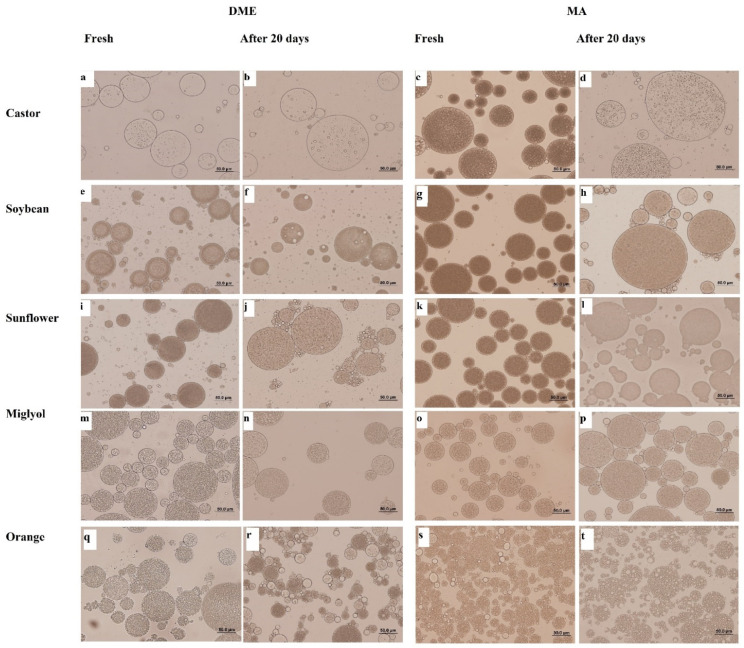
Optical microscopy images of fresh DEs prepared by membrane emulsification (**a**,**e**,**i**,**m**,**q**), DEs prepared by membrane emulsification after 20 days of storage (**b**,**f**,**j**,**n**,**r**), fresh DEs prepared by mechanical agitation (**c**,**g**,**k**,**o**,**s**) and DEs prepared by mechanical agitation after 20 days of storage (**d**,**h**,**l**,**p**,**t**). All scale bars represent 50 µm.

In Figure 3, the optical microscopy images of W_1_/O/W_2_ DEs formulated and prepared both by DME and MA are shown.

The presence of inner water droplets can be observed in all the images, confirming the formation of DEs, in correlation with the mean droplet sizes provided by the Mastersizer equipment.

As a general trend, variations in droplet size with time were observed when MA was used for DE preparation, whereas droplet size variation was not found during the 20 days for DE prepared by DME. Similar behaviors have been reported in other studies where DEs have been prepared by MA and DME by Matos et al. [27,28].

The largest differences in droplet size with time were observed in DEs formulated with castor oil prepared by MA, where a remarkable increase in size was observed with time. This increase in size was also found, but less noticeable, in DEs where soybean and sunflower oils were used as the oil phase. It is important to take into account that these three oils were the ones that showed larger viscosity (Table 1). On the contrary, DEs prepared with orange oil, the oil with the lowest viscosity, showed the opposite behavior, presenting a clear reduction in droplet size over time, especially in the case when DEs were prepared by MA.

**Figure 4:**
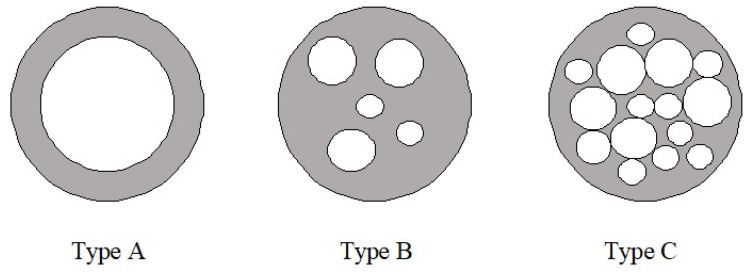
Classification of types of emulsions according to the distribution of the oil phase.

Florence and Whitehill [46] classified DEs into three different types depending on the disposition of the water droplets inside the oil drops. Figure 4 shows schematically the three types described: type A represents a small portion of water drops inside the oil drop (generally one drop); type B consists of oil drops with more numerous internal droplets but with smaller size that the ones observed in type A, while type C presents a large amount of water droplets inside the oil drop, making it even more difficult to identify each water droplet. All the DEs prepared showed a type C structure, where tiny and numerous aqueous droplets could be appreciated inside the identified oil drops. However, DEs prepared with castor oil showed a lower concentration of internal water drops when they were prepared by MA, probably due to their large viscosity, which should require large energy to produce internal aqueous droplets in that high viscosity oil. The lower concentration of water droplets after 20 days was also appreciable when DEs with castor oil were prepared by DME. These results are consistent with the smallest change in droplet size and polydispersity for the rest of the oils used, which could be attributed to the significantly lower viscosities of the oils used [38,39,46,47].

### 3.4. Stability

The double droplets of the W_1_/O tend to migrate to the upper zone of the cell that contains the DEs due to their low density compared to the continuous phase (W_2_). The creaming velocity, which will be defined by Stoke’s Law, can be influenced by the viscosity and phases densities and the diameter of the dispersed phase drops, as observed by Matos et al. in previous works [29].

**Figure 5:**
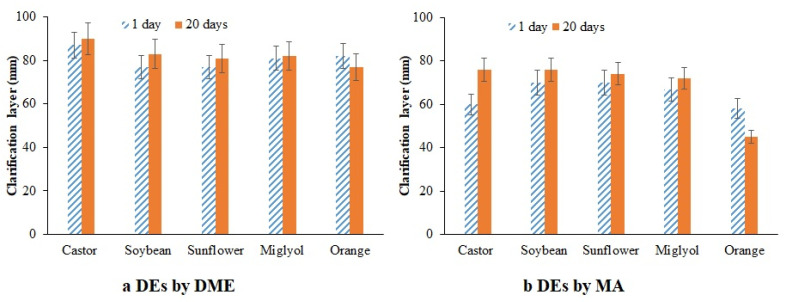
Clarification layer after 1 day and after 20 days of storage. (**a**) DEs prepared by DME; (**b**) DEs prepared by MA.

As a general trend, no variations on droplet size were observed on the backscattering profiles measured by Turbiscan equipment. However, all the samples showed a large clarification at the bottom of the cell. The clarification layer obtained after 20 days of storage was generally larger in DEs prepared by DME than in the case of DEs prepared by MA. The percentage of the clarification layer for DEs prepared by MA was in the range 72–76% of the whole sample, with the exception of DEs with orange oil as the oil phase, where the clarification layer after 20 storage days comprised only 45% of the whole sample (Figure 5a). However, for DEs prepared by DME, the thickness of the clarification layer of all samples ranged from 90 to 77% (Figure 5b).

The change of the thickness in the clarified layer indicates that destabilization could be taking place by droplets coalescence, Ostwald ripening phenomena or other phenomena involved on the swelling and deswelling of the inner droplets thought the oil drop [29,44]. It is important to point out that the clarification layer was increased with time, as was expected since drops tend to migrate to the surface due to their lower density, as was observed in other works [41]. However, this was not the case of DEs with orange oil as the oil phase. The clarification layer of these DEs prepared by DME suffered a slight reduction, and this phenomenon was more noticeable when the same DEs were prepared by MA, where the clarification layer decreased from 58 to 45%. The only explanation found for this behavior is the swelling of the W_2_ phase that moves through the oil drop, increasing the concentration of water droplets inside the oil drops. This phenomenon was frequently observed in several works where DEs were formulated, usually promoted by differences in osmotic pressure between both aqueous phases or differences in concentration of some compounds, as described by Khadem et al. and observed by Díaz-Ruíz et al. [44,45]. In this case, the different concentration promoted by the presence of pomegranate in the W_1_ phase will enhance the swelling of the W_2_ to the W_1_. The fact that this behavior was only noticeable in DEs with orange oil as the oil phase could be related to the low viscosity of this oil, being that this is the only case in which the driving force produced by the difference in concentration could be enough to produce oil drop rupture and penetration of it.

**Figure 6:**
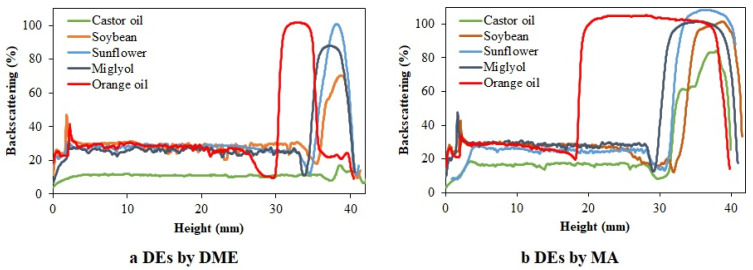
Backscattering profiles of DEs after 20 days of storage. (**a**) DEs prepared by DME; (**b**) DEs prepared by MA.

Figure 6 shows the backscattering profiles of the samples after 20 days of storage. At the left part of each graph, it can be easily noticeable that all the samples showed a clarification layer, indicated by the low value of backscattering in this area. The DEs formulated with orange oil where the ones that showed a lower range of clarification layer. Moreover, a large instability of DEs prepared with castor oil was also observed, even when no emulsion was observed when DME was used as preparation method, and large irregularities on the emulsion layer were found when this DE was prepared by MA. This phenomenon indicates that some coalescence could be taking place, producing a nonhomogenous emulsion layer, which was also observed for DEs produced with DME when soybean oil was used as the oil phase, since these were the two oils with larger viscosity values. Moreover, according to Figure 6a, where DME was used as preparation method, an oil layer at the top of the sample when orange oil was used, indicated by a large reduction of the backscattering value at the area corresponding to the top of the cell, can be observed. It is important to point out that flavor oils, frequently used in food and beverages, as it is the case of orange oil, had lower density compared to other mineral and vegetable oils. This flavor oil is more likely to suffer creaming phenomena due to the large density difference with the aqueous phase. Moreover, its large water solubility compared to other oils enhance Ostwald ripening destabilizing phenomena since small oil drops can easily be dissolved in water [48]. However, this type of oil offers a good alternative for preparing emulsions, which encapsulated some compounds with intermediate solubility in water and oil, as observed by Matos et al. [49]. Several works were completed in the last decade in which different types of triglycerides are added to flavor oils to inhibit Ostwald ripening phenomena by McClements et al. [50] and Park et al. [51].

The results indicated that intermediate oil viscosities are more appropriate to ensure the stability of the large DEs, avoiding coalescence and swelling phenomena.

### 3.5. Encapsulation Efficiency (EE)

**Figure 7:**
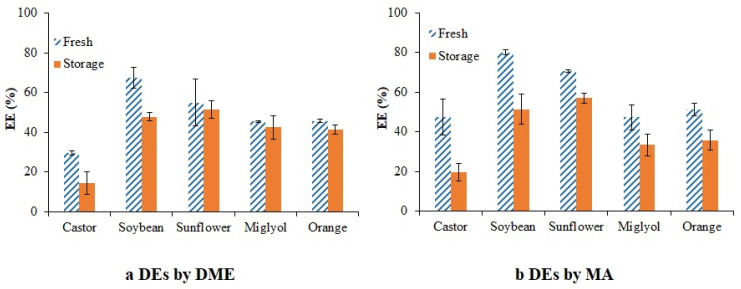
Encapsulation efficiency of PPE in DEs, fresh and after 20 days of storage. (**a**) Prepared by DME; (**b**) Prepared by MA.

Figure 7 shows the values of EE for DEs prepared by DME and MA methods, both fresh and after 20 days of storage.

As a general trend for DEs prepared with high viscosity oils, higher EE values were obtained when MA was used as the preparation method, but no differences between preparation methods were observed when low viscosity oils were used for DE formulation. However, only when castor oil was used, the one with the highest viscosity, low EE values were observed for both preparation methods (EE values around 30–50%). The use of oils with intermediate viscosity values gave higher EE in the range 80–70% when MA was used, while values around 70–50% were recorded for DEs produced by DME. DEs formulated with soybean oil showed the highest EE values in both cases (80% for MA DEs and 68% for DME DEs).

In all cases, the EE values after 20 days of storage were lower than the ones registered for the same emulsions when they were fresh. This indicates the release of the PPE to the external aqueous phase. Two mechanisms have been described by Lambda et al. and Pays et al. [42,52] to explain the release of a chemical substance in DEs: the deswelling of the inner phase and the diffusion and/or permeation of the chemical substance across the oily intermediate phase reaching the external aqueous phase. The large release observed for the DEs formulated using castor oil produced by MA could be attributed to the high size variations and large instability found when this oil was used. Despite the higher EE values obtained by MA, the greatest release was observed for these samples, in accordance with the larger polydispersity observed in these emulsions (Table 2 and Figure S1, supplementary material), which could involve different velocities in the biocompounds’ release. In general, those DEs that were more stable in relation to droplet size showed a larger homogeneity and lower biocompound release (DEs produced by DME using sunflower oil and Miglyol), in agreement with some studies by Matos et al., Charcosset et al. and Okochi et al. [27,53,54] in which trans-resveratrol was encapsulated in DEs. Even the swelling of DEs formulated by orange oil with lower PPE release was observed, indicating that the swelling phenomena was mainly related to the migration of W_1_ to W_2_ without producing migration of the biocompound encapsulated.

## 4. Conclusions

PPE was efficiently encapsulated in W_1_/O/W_2_ DEs. The encapsulation depended on the method of DE preparation used and the forces involved during the emulsification process. The DME technique led to more homogenous emulsions but with lower EE values than ones obtained by MA. However, the use of MA involved larger release values.

The DEs with better stability, lower particle size and higher EE and low release were those prepared using oils with viscosities around 0.006–0.030 Pa·s, such as Miglyol or sunflower oil. DEs formulated with sunflower oil, prepared by DME, could be considered as suitable systems to encapsulate punicalagin with concentrations up to 11,000 mg/L of PPE.

DME were evaluated to be a suitable technique to obtain DEs with desired drop size. The size of the droplets can be easily controlled with this method by the diameter of the membrane pores and also by the operation parameters. These parameters will determine the values of the main forces responsible for droplet formation and their detachment from the membrane surface. DEs prepared by DME presented high stability, high EE and low biocompound release during storage.

## Figures and Tables

**Table 1:** Viscosities of the oils.

Oil	Viscosity (Pa·s) X ± SD
Castor	0.517 ± 0.004 ^d^
Soybean	0.055 ± 0.009 ^c^
Sunflower	0.029 ± 0.007 ^b^
Miglyol	0.006 ± 0.004 ^a^
Orange	0.005 ± 0.004 ^a^

X: mean; SD: standard deviation. Letters (^a–d^) indicate significant differences between oil viscosities.

**Table 2:** Span value of DEs (W1/O/W2) with PPE encapsulated of one day and after storage (20 days) and main droplet size in DE fresh and after 20 days of storage.

Oil	DME				MA			
	Span (X ± SD)	MDS (µm)	Span (X ± SD)	MDS (µm)	Span (X ± SD)	MDS (µm)	Span (X ± SD)	MDS (µm)
	Fresh		20 Days		Fresh		20 Days	
Castor	2.7 ± 0.4 ^b,Y,w^	56.23	2.8 ± 0.5 ^a,X,w^	103.6	2.7 ± 0.3 ^b,X,w^	103.6	1.8 ± 0.2 ^a,X,z^	190.8
Soybean	2.2 ± 0.2 ^ab,Y,w^	48.13	2.5 ± 0.2 ^a,X,w^	103.6	1.73 ± 0.01 ^a,X,w^	76.3	2.7 ± 0.9 ^ab,X,z^	120.7
Sunflower	2.1 ± 0.1 ^ab,Y,w^	56.23	2.3 ± 0.5 ^a,X,w^	120.7	1.64 ± 0.04 ^a,X,w^	76.3	3.5 ± 0.6 ^b,X,z^	76.3
Miglyol	1.9 ± 0.1 ^ab,Y,w^	65.5	2.1 ± 0.4 ^a,X,w^	76.3	1.5 ± 0.1 ^a,X,w^	65.5	2.2 ± 0.2 ^ab,X,z^	65.5
Orange	1.6 ± 0.1 ^a,Y,w^	56.23	2.2 ± 0.1 ^a,X,w^	41.4	1.80 ± 0.03 ^a,X,w^	19.3	2.2 ± 0.2 ^ab,X,z^	26.2

X: mean; SD: standard deviation. Letters (^a,b^) indicate significant differences between oils on the same day for each method, letters (^X,Y^) indicate significant differences on the same day between methods, letters (^w,z^) indicate significant differences on the same method in the storage (1–20 days). DME: direct membrane emulsification; MA: mechanical agitation; MDS: mean droplet size (DE fresh and after 20 days of storage).

## Data Availability

Data is contained within the article (or Supplementary Material).

## Supplementary Materials

The following supporting information can be downloaded at: https://www.mdpi.com/article/10.3390/foods11030310/s1, Figure S1: Droplet size distribution prepared by DME and MA in fresh DEs (1 day) and in storage (20 days).

## References

[1] Akhtar S., Ismail T., Fraternale D., Sestili P. (2015). Pomegranate peel and peel extracts: Chemistry and food features. Food Chem..

[2] García P., Fredes C., Cea I., Lozano-Sánchez J., Leyva-Jiménez F., Robert P., Vergara C., Jimenez P. (2021). Recovery of Bioactive Compounds from Pomegranate (*Punica granatum* L.) Peel Using Pressurized Liquid Extraction. Foods.

[3] Gustavsson J., Cederberg C., Sonesson U., van Otterdijk R., Meybeck A. (2011). Global Food Losses and Food Waste: Extent, Causes and Prevention.

[4] United Nations Sustainable Consumption and Production. United Nations Sustainable Development. https://www.un.org/sustainabledevelopment/sustainable-consumption-production/.

[5] Fischer U.A., Carle R., Kammerer D.R. (2011). Identification and quantification of phenolic compounds from pomegranate (*Punica granatum* L.) peel, mesocarp, aril and differently produced juices by HPLC-DAD–ESI/MSn. Food Chem..

[6] Çam M., Hışıl Y. (2010). Pressurised water extraction of polyphenols from pomegranate peels. Food Chem..

[7] Kaderides K., Goula A.M., Adamopoulos K.G. (2015). A process for turning pomegranate peels into a valuable food ingredient using ultrasound-assisted extraction and encapsulation. Innov. Food Sci. Emerg. Technol..

[8] Quattrucci A., Ovidi E., Tiezzi A., Vinciguerra V., Balestra G.M. (2013). Biological control of tomato bacterial speck using Punica gran-atum fruit peel extract. Crop Prot..

[9] Reddy M., Gupta S., Jacob M., Khan S., Ferreira D. (2007). Antioxidant, Antimalarial and Antimicrobial Activities of Tannin-Rich Frac-tions, Ellagitannins and Phenolic Acids from *Punica granatum* L.. Planta Med..

[10] Viuda-Martos M., Ruiz-Navajas Y., Fernández-López J., Sendra E., Sayas-Barberá E., Pérez-Álvarez J.A. (2011). Antioxidant properties of pomegranate (*Punica granatum* L.) bagasses obtained as co-product in the juice extraction. Food Res. Int..

[11] Kharchoufi S., Licciardello F., Siracusa L., Muratore G., Hamdi M., Restuccia C. (2018). Antimicrobial and antioxidant features of ‘Gabsi’ pomegranate peel extracts. Ind. Crops Prod..

[12] Molva C., Baysal A.H. (2015). Evaluation of bioactivity of pomegranate fruit extract against Alicyclobacillus acidoterrestris DSM 3922 vegetative cells and spores in apple juice. LWT Food Sci. Technol..

[13] Çam M., Içyer N.C., Erdoğan F. (2014). Pomegranate peel phenolics: Microencapsulation, storage stability and potential ingredient for functional food development. LWT Food Sci. Technol..

[14] Comunian T.A., da Silva Anthero A.G., Bezerra E.O., Moraes I.C.F., Hubinger M.D. (2020). Encapsulation of Pomegranate Seed Oil by Emulsification Followed by Spray Drying: Evaluation of Different Biopolymers and Their Effect on Particle Properties. Food Bioprocess Technol..

[15] Endo E.H., Ueda-Nakamura T., Nakamura C.V., Filho B.P.D. (2012). Activity of Spray-dried Microparticles Containing Pomegranate Peel Extract against Candida albicans. Molecules.

[16] Goula A., Adamopoulos K. (2012). A method for pomegranate seed application in food industries: Seed oil encapsulation. Food Bioprod. Process..

[17] Robert P., Gorena T., Romero N., Sepulveda E., Chavez J., Saenz C. (2010). Encapsulation of polyphenols and anthocyanins from pomegranate (*Punica granatum*) by spray drying: Encapsulation of polyphenols and anthocyanins. Int. J. Food Sci. Technol..

[18] Zam W., Ruiz J.C.R., Campos M.R.S. (2017). Fortification of dairy products by microcapsules of polyphenols extracted from pomegranate peels. New Polymers for Encapsulation of Nutraceutical Compounds.

[19] Zam W., Bashour G., Abdelwahed W., Khayata W. (2014). Alginate-pomegranate peels’ polyphenols beads: Effects of formulation parameters on loading efficiency. Braz. J. Pharm. Sci..

[20] Heidari F., Jafari S.M., Ziaiifar A.M., Malekjani N. (2022). Stability and release mechanisms of double emulsions loaded with bioactive compounds; a critical review. Adv. Colloid Interface Sci..

[21] Robert P., Zamorano M., González E., Silva-Weiss A., Cofrades S., Giménez B. (2019). Double emulsions with olive leaves extract as fat replacers in meat systems with high oxidative stability. Food Res. Int..

[22] Van der Graaf S., Schroën C., Boom R.M. (2005). Preparation of double emulsions by membrane emulsification—A review. J. Membr. Sci..

[23] Garti N. (1997). Progress in Stabilization and Transport Phenomena of Double Emulsions in Food Applications. LWT Food Sci. Technol..

[24] Berendsen R., Güell C., Henry O., Ferrando M. (2014). Premix membrane emulsification to produce oil-in-water emulsions stabilized with various interfacial structures of whey protein and carboxymethyl cellulose. Food Hydrocoll..

[25] Joscelyne S.M., Trägårdh G. (2000). Membrane emulsification—A literature review. J. Membr. Sci..

[26] Anarakdim K., Gutiérrez G., Cambiella Á., Senhadji-Kebiche O., Matos M. (2020). The Effect of Emulsifiers on the Emulsion Stability and Extraction Efficiency of Cr(VI) Using Emulsion Liquid Membranes (ELMs) Formulated with a Green Solvent. Membranes.

[27] Matos M., Gutiérrez G., Coca J., Pazos C. (2014). Preparation of water-in-oil-in-water (W1/O/W2) double emulsions containing trans-resveratrol. Colloids Surf. A Physicochem. Eng. Asp..

[28] Matos M., Gutiérrez G., Iglesias O., Coca J., Pazos C. (2015). Enhancing encapsulation efficiency of food-grade double emulsions con-taining resveratrol or vitamin B12 by membrane emulsification. J. Food Eng..

[29] Matos M., Gutiérrez G., Martínez-Rey L., Iglesias O., Pazos C. (2018). Encapsulation of resveratrol using food-grade concentrated double emulsions: Emulsion characterization and rheological behaviour. J. Food Eng..

[30] Berendsen R., Güell C., Ferrando M. (2015). A procyanidin-rich extract encapsulated in water-in-oil-in-water emulsions produced by premix membrane emulsification. Food Hydrocoll..

[31] Suárez M.A., Gutiérrez G., Coca J., Pazos C. (2013). Geometric parameters influencing production of O/W emulsions using flat metallic membranes and scale-up. J. Membr. Sci..

[32] Gutiérrez G., Matos M., Benito J.M., Coca J., Pazos C. (2014). Preparation of HIPEs with controlled droplet size containing lutein. Colloids Surf. A Physicochem. Eng. Asp..

[33] Matos M., Gutiérrez G., Lobo A., Coca J., Pazos C., Benito J.M. (2016). Surfactant effect on the ultrafiltration of oil-in-water emulsions using ceramic membranes. J. Membr. Sci..

[34] Evans J., Hammond E., Gigiel A., Fostera A., Reinholdt L., Fikiin K., Zilio C. (2014). Assessment of methods to reduce the energy consumption of food cold stores. Appl. Therm. Eng..

[35] Glazer I., Masaphy S., Marciano P., Bar-Ilan I., Holland D., Kerem Z., Amir R. (2012). Partial Identification of Antifungal Compounds from *Punica granatum* Peel Extracts. J. Agric. Food Chem..

[36] Silva W., Torres-Gatica M.F., Oyarzun-Ampuero F., Silva-Weiss A., Robert P., Cofrades S., Giménez B. (2018). Double emulsions as potential fat replacers with gallic acid and quercetin nanoemulsions in the aqueous phases. Food Chem..

[37] Robert P., Vergara C., Silva-Weiss A., Osorio F.A., Santander R., Sáenz C., Giménez B. (2020). Influence of gelation on the retention of purple cactus pear extract in microencapsulated double emulsions. PLoS ONE.

[38] Omotosho J.A., Whateley T.L., Law T.K., Florence A.T. (1986). The Nature of the Oil Phase and the Release of Solutes from Multiple (w/o/w) Emulsions. J. Pharm. Pharmacol..

[39] Raynal S., Grossiord J.L., Seiller M., Clausse D. (1993). A topical W/O/W multiple emulsion containing several active substances: For-mulation, characterization and study of release. J. Controlled Release.

[40] Smułek W., Siejak P., Fathordoobady F., Masewicz Ł., Guo Y., Jarzębska M., Kitts D.D., Kowalczewski P.Ł., Baranowska H.M., Stangierski J. (2021). Whey Proteins as a Potential Co-Surfactant with *Aesculus hippocastanum* L. as a Stabilizer in Nanoemulsions Derived from Hempseed Oil. Molecules.

[41] Jarzębski M., Smułek W., Siejak P., Rezler R., Pawlicz J., Trzeciak T., Jarzębska M., Majchrzak O., Kaczorek E., Kazemian P. (2021). *Aesculus hippocastanum* L. as a Stabilizer in Hemp Seed Oil Nanoemulsions for Potential Biomedical and Food Applications. Int. J. Mol. Sci..

[42] Lamba H., Sathish K., Sabikhi L. (2015). Double Emulsions: Emerging Delivery System for Plant Bioactives. Food Bioprocess Technol..

[43] Yuan Q., Aryanti N., Gutiérrez G., Williams R.A. (2009). Enhancing the Throughput of Membrane Emulsification Techniques to Manufacture Functional Particles. Ind. Eng. Chem. Res..

[44] Díaz-Ruiz R., Valdeón I., Álvarez J.R., Matos M., Gutiérrez G. (2021). Simultaneous encapsulation of trans-resveratrol and vitamin D3 in highly concentrated double emulsions. J. Sci. Food Agric..

[45] Khadem B., Khellaf M., Sheibat-Othman N. (2020). Investigating swelling-breakdown in double emulsions. Colloids Surf. Physicochem. Eng. Asp..

[46] Florence A.T., Whitehill D. (1981). Some features of breakdown in water-in-oil-in-water multiple emulsions. J. Colloid Interface Sci..

[47] Florence A.T., Whitehill D., Shah D.O. (1985). Stability and Stabilization of Water-in-Oil-in-Water Multiple Emulsions. Macro- and Microemulsions.

[48] Park J., Lee J., McClements D.J., Choi S.J. (2020). Inhibition of Droplet Growth in Model Beverage Emulsions Stabilized Using Poly (ethylene glycol) Alkyl Ether Surfactants Having Various Hydrophilic Head Sizes: Impact of Ester Gum. Appl. Sci..

[49] Matos M., Marefati A., Barrero P., Rayner M., Gutiérrez G. (2021). Resveratrol loaded Pickering emulsions stabilized by OSA modified rice starch granules. Food Res. Int..

[50] McClements D., Henson L., Popplewell L.M., Decker E.A., Choi S.J. (2012). Inhibition of Ostwald Ripening in Model Beverage Emulsions by Addition of Poorly Water Soluble Triglyceride Oils. J. Food Sci..

[51] Park S.H., Hong C.R., Choi S.J. (2020). Prevention of Ostwald ripening in orange oil emulsions: Impact of surfactant type and Ostwald ripening inhibitor type. LWT.

[52] Pays K. (2002). Double emulsions: How does release occur?. J. Control. Release.

[53] Charcosset C. (2009). Preparation of emulsions and particles by membrane emulsification for the food processing industry. J. Food Eng..

[54] Okochi H., Nakano M. (1997). Comparative Study of Two Preparation Methods of w/o/w Emulsions: Stirring and Membrane Emulsi-fication. Chem. Pharm. Bull..

