# Supplementary material for: Encapsulation of Pomegranate Peel Extract (Punica granatum L.) by Double Emulsions: Effect of the Encapsulation Method and Oil Phase
Source: Foods. 2022 Jan 24;11(3):310. doi: 10.3390/foods11030310 (PMC8833941; doi:10.3390/foods11030310)
Supplement: Supplementary file 1 [file foods-11-00310-s001.zip › foods-1529813-supplementary.pdf]

Supplementary Information

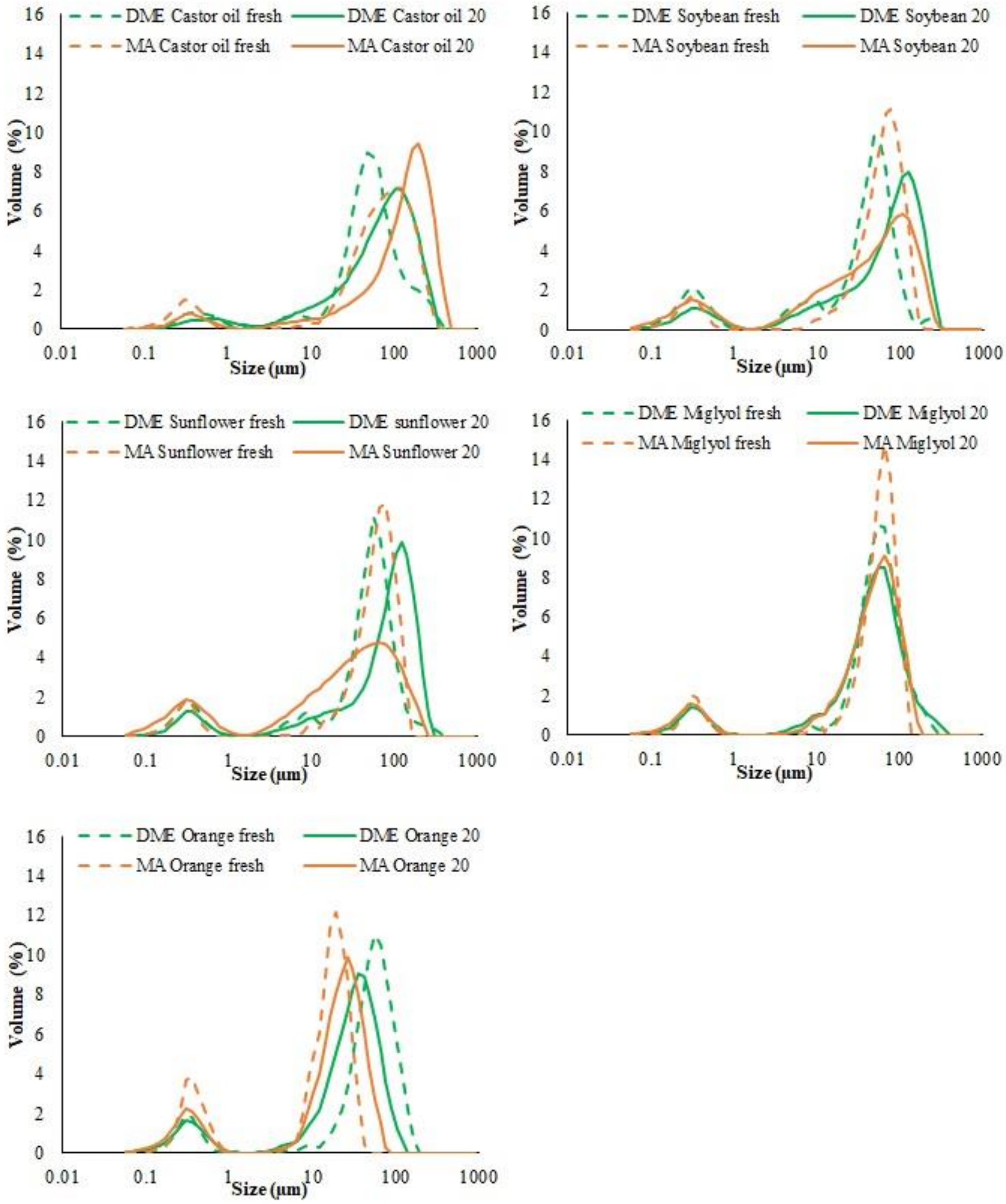

Figure S1. Droplet size distribution prepared by DME and MA in fresh DEs (1 day) and in storage (20 days).
